# Suicide prevention and education

**DOI:** 10.3389/fpubh.2026.1935894

**Published:** 2026-08-05

**Authors:** Brittany Faith Thacker

**Affiliations:** Independent Researcher, Pikeville, KY, United States

**Keywords:** antecedent, behavior analysis, education, prevention, suicide

## Introduction

The Centers for Disease Control and Prevention (CDC) website states, “Suicide is death caused by injuring oneself with the intent to die” (1). With this definition there must be an act that leads to the actual death of the individual with that being the individual's goal when performing that specific behavior. Any accidental or unintentional deaths are not included within the definition for suicide.

Also, according to the CDC, “A suicide attempt is when someone harms themselves with any intent to end their life, but they do not die as a result of their actions” (1). This would suggest that an action took place to end one's own life but did not achieve the intended purpose. Prevention and education organizations also define suicide in terms of impact on the community. A blurb on Save.org states, “Suicide is a complex public health issue that requires coordination and cooperation among health care providers, individuals and family members, and treatment services and the community” (2). It is important to consider the global impact suicide has on communities and lives beyond those who initiate their own end of life. There are many factors that come into play once an attempt or success occurs.

## Research/statistics overview

Many people have dealt either directly or indirectly with suicide. “As many as 40%−50% of the population have been exposed to suicide in their lifetime” (3). This number can include those who have made attempts or are dealing with the consequences of a loved one's suicide behavior. There are many actions that must be taken by loved ones after a suicide has occurred. In addition to the mental health concerns that are newly present (grief, depression, survivor's guilt), they also must serve as estate administrators and complete legal obligations that are left at the end of one's life.

Suicide is prevalent among all ages and cultures. A concerning aspect of this is the number of young people that are directly impacted by suicide and suicide attempts. While there are general assumptions that the youth of first world nations would not experience life-threatening stressors, the statistics would disagree with that notion. The American Association of suicidology states, “Suicide ranks as the 10th leading cause of death in the US; It ranks as the 2nd leading cause of death for 15–24-year-olds” (4). For this population, suicide directly comes behind unintentional deaths such as accidental overdoses. This information calls into question the reasons behind such a large prevalence of suicide among this young age group and the need for more effective resources in prevention and education.

Also, according to the American association of suicidology, “1 person every 11.1 min killed themselves” (4). This is an alarming rate that will have an unmediated effect on the world's population totals. This is a steady rate of the human population ending their own lives. As suicide exists among all age groups and cultural subcategories, there is an implicit influence on the overall global impact.

The Center for Disease Control and Prevention states, “Suicide is highly prevalent. Suicide presents a major challenge to public health in the United States and worldwide. It contributes to premature death, morbidity, lost productivity, and health care costs” (1).

The World Health Organization (5) furthered explains that the number of individuals who die by suicide is over 720,000 per year and “Seventy-three per cent of global suicides occur in low- and middle-income countries.”

“The good news is that more than 90% of people who attempt suicide and survive never go on to die by suicide” (1). This is an encouraging statistic. The percentage suggests that post-suicide attempt resources are effective in reducing risk factors for suicide behavior and/or treating underlying co-morbid issues that lead to the suicide attempt behavior.

This is an opinion article written to address a public health and safety concern and promote global awareness toward individuals finding techniques and treatment options. This article contains information gathered from studies conducted in the United States, however statistics included and the discussed techniques have the intended aim to be generalized to the world-wide public.

## Treatment/intervention

“Suicide is a serious public health problem that can have lasting harmful effects on individuals, families, and communities. There are many factors that contribute to suicide. The goal of suicide prevention is to reduce factors that increase risk and increase factors that promote resilience” (1). Since there are so many factors that can contribute to suicide and suicide attempts, it might be advantageous to look at intervening on the behavior of suicide and suicide attempts rather than every factor that can lead up to the decision to end one's life.

Due to this piece being conducted through a behavior analytic lens, strategies discussed are aimed at an individual approach to treatment and symptom management.

## Behavior intervention plan

As suicide can be defined as a behavior (the action of taking one's own life with the intent of doing so), having a behavior intervention plan is a plausible treatment approach. This approach would include antecedent interventions that would prevent or lessen the opportunity for the event to take place. There could also be consequence strategies included for suicide attempts (any thoughts of self-harm behaviors that could potential and are done with the implicit purpose of ending one's own life) that would reduce the likeliness of any future occurrences of attempt behavior(s).

## Antecedent strategies

Possible antecedent strategies could include therapy sessions and developing a support network. “Treatments that have demonstrated effectiveness in reducing suicidal behavior are cognitive behavior therapy for depression and dialectical behavior therapy for borderline personality disorder” (6). These suggested therapies would work well for individuals who have comorbid or underlying mental health diagnosis. A recent study on the use of Cognitive Behavior Therapy for suicide prevention (CBT-SP) concluded, “CBT-SP is an empirically supported treatment modality that reduces suicidal behaviors by 50% or more when delivered in outpatient mental healthcare settings. The treatment's efficacy is attributable in part to its use of procedures that focus on reducing two core vulnerabilities for suicidal behavior: emotion dysregulation and cognitive BRYAN 255 inflexibility” (7).

“Dialectical behavior therapy (DBT) prioritizes suicidal behavior and other self-directed violence as the primary treatment targets and has been demonstrated to reduce self-directed violence in clinical trials” (8). This approach expands on the principles of cognitive behavior therapy and attempts to modify targeted behavior(s) through emotional regulation and skill improvement strategies.

A support network can be made up of relatives or friends who are aware of possible suicidal ideation and can assist in watching for warning signs of the behavior. They can also be used in the event of a trigger to call upon to talk through current and immediate plans and give support to work through any challenging thoughts.

Also, learning about resources and strategies prior to immediate suicide behavior can have a very real impact on the actual occurrence of the behavior. Learning coping skills techniques such as mediation, thought redirection, and breathing exercises can help reduce stress in specific moments and give clarity to potential life-threatening behavior (9).

## Consequence strategies

Possible consequence strategies to suicide attempts may include access to readily available resources and learned coping skills. Crisis resources are maintained by many suicide prevention organizations throughout the world for those who have immediate intent to end their own lives. These are available to all people, regardless of if there has been a one-time trigger for suicidal behavior or if there are comorbid factors such as mental health diagnosis that are impacting the behavior. Resources such as 24/7 phone hotlines, live text opportunities and chatrooms are continuously maintained for anyone reaching out for help while experiencing suicide or suicide attempt behavior.

## Need for further research

“Despite national goals to lower the suicide rate, several recent reports have documented a steady increase in suicide rates in recent years. From 1999 through 2018, suicide rates increased for both males and females, with greater increases occurring after 2006” (10). This stands to point that there is a pandemic occurring in the mental health field that only continues to worsen over time. Rates have not lessened, but rather increases have been seen among all subgroups, even though there are a plethora of resources available at instant via the internet.

As there are so many people impacted by suicide on an hourly basis, there is a clear need for further research and development on prevention and intervention approaches.

Many behavior interventions could be utilized in the prevention of suicide and attempts. The implementation of applied behavior analysis, cognitive behavior therapy, dialectical behavior therapy, and other behavior approaches in suicide prevention could help lessen the rates of suicide and suicide attempts among the human population. Research needs to be done on specific treatments and interventions that would work best for sub-groups including age ranges, medical diagnoses, cultures, and prior history of impact by suicide. These studies could be conducted by using many measures and by multiple organizations to ensure fidelity and validity of results. There should also be a multi-practitioner approach model as the impacts, implications, and triggers of suicide and suicide attempt behaviors span across disciplines. This is an international and far-reaching behavior in such that the prevention and treatment process can include everyone on the planet.
